# Editorial: Brain pathology and rehabilitation mechanisms of neuromodulation in psychiatric disorders

**DOI:** 10.3389/fpsyt.2025.1620700

**Published:** 2025-05-21

**Authors:** Mi Yang, Zezhi Li, Dechao Feng

**Affiliations:** ^1^ Department of Scientific Research Management, The Fourth People’s Hospital of Chengdu, Chengdu, China; ^2^ The Clinical Hospital of Chengdu Brain Science Institute, Ministry of Education (MOE) Key Lab for Neuroinformation, University of Electronic Science and Technology of China, Chengdu, Sichuan, China; ^3^ School of Life Science and Technology, University of Electronic Science and Technology of China, Chengdu, China; ^4^ Department of Psychiatry, The Affiliated Brain Hospital of Guangzhou Medical University, Guangzhou, China; ^5^ Department of Psychiatry, Guangdong Engineering Technology Research Center for Translational Medicine of Mental Disorders, Guangzhou, China; ^6^ Department of Urology, Institute of Urology, West China Hospital, Sichuan University, Chengdu, China

**Keywords:** brain pathology, neuropathological mechanism, neuromodulation, psychiatric disorders, non-invasive brain stimulation (NIBS), rehabilitation therapy, transcranial magnetic stimulation (TMS), transcranial electrical stimulation (TES)

The hallmark characteristics of mental illnesses manifest in substantial abnormalities in an individual’s perception of reality, emotional regulation, and social functioning, exerting a profound impact on psychological well-being and quality of life. Among the aforementioned patients, those afflicted with severe mental illnesses, including but not limited to bipolar disorder and schizophrenia, are subject to an exacerbated physical and mental burden (1). The etiology of mental illnesses may be underpinned by genetic factors (2). Environmental stress and psychological trauma have been identified as crucial triggers for the onset of pathological processes. These processes result in cognitive dysfunction and behavioral disorders through pathways involving neurotransmitters (e.g., dopamine, serotonin, acetylcholine), brain structures (e.g., cortical thickness, white matter connectivity), and epigenetics (e.g., DNA methylation). However, our understanding of the neurobiological mechanisms underlying these diseases remains incomplete, resulting in limited efficacy of conventional drug treatments in improving patients’ cognitive functions and clinical symptoms. Moreover, these treatments frequently entail deleterious side effects, including obesity and metabolic abnormalities, which induce great challenges to clinical treatment and health care. Consequently, an exhaustive investigation into the etiology of mental diseases, in conjunction with the exploration of diverse therapeutic modalities, is imperative to furnish substantial empirical evidence for the precise diagnosis and customized treatment of mental illnesses.

This Research Topic focuses on examining the efficacy and pathological mechanisms of neuroregulation methods in the rehabilitation treatment of mental illnesses. It includes five studies, primarily addressing schizophrenia and depression, involving Continuous theta burst stimulation (cTBS) therapy, paradigm exploration. The following is a brief introduction to these research findings.

## Schizophrenia-related research

Schizophrenia-related psychosis risk syndrome (PRS) is characterized by the protracted prodromal symptoms exhibited by patients preceding their inaugural episode. However, the neurobiological mechanisms underlying this syndrome remain unclear. Ruan et al. employed a representational similarity analysis (RSA) approach to examine the neuropsychological differences between PRS patients and healthy individuals. The study revealed that, under both 5 Hz and 10 Hz conditions, the functional coupling between steady-state visual evoked potentials (SSVEPs) was diminished in the fusiform region for patients with psychic disorders (PRS). Furthermore, the activation in the visual regions associated with 10 Hz SSVEP and emotional matching was also diminished. These findings suggest that individuals with PRS exhibit early-stage visual processing abnormalities, which may provide important insights for the early and precise diagnosis of schizophrenia.

The cTBS has emerged as a significant treatment modality for auditory hallucinations in patients diagnosed with schizophrenia. Ye et al. conducted a meta-analysis with the objective of evaluating the efficacy of cTBS in the treatment of auditory hallucinations. Four randomized controlled trials (RCTs) encompassing a total of 151 patients diagnosed with auditory hallucinations was conducted. The findings suggested that cTBS did not demonstrate a substantial advantage over sham stimulation in addressing hallucinations. This discrepancy may be attributable to the limited sample size and the substantial heterogeneity among the studies. However, patients who underwent more than 10 sessions of cTBS demonstrated significant improvement in their auditory hallucination symptoms. This finding indicates that patients receiving cTBS for auditory hallucinations may necessitate prolonged treatment, although further experimental research is necessary to ascertain the most efficacious cTBS stimulation protocol for addressing auditory hallucinations.

## Depression-related research

Depression is among the most prevalent mental disorders, resulting in a significant societal and familial burden. Conventional pharmaceutical remedies frequently entail adverse effects, thereby prompting researchers to actively pursue alternative therapeutic modalities. Among these, exercise therapy has demonstrated considerable potential for the prevention and treatment of depression. Xue et al. discovered that the efficacy of exercise interventions in ameliorating depression varies across different age demographics. For instance, in adolescents, exercise exerts a primary influence on brain development and hormonal regulation. In middle-aged individuals, its impact is focused on the HPA axis and cardiovascular health. In the elderly, the emphasis shifts to immune regulation and social support. These findings corroborate the age-dependent nature of exercise therapy in treating depression, thereby establishing a clinical foundation for personalized exercise interventions for depression.

Imbert et al. developed a randomized, double-blind, controlled, dual-intervention treatment plan for patients diagnosed with major depressive disorder (MDD). This treatment plan specifically targets anhedonia by combining repetitive transcranial magnetic stimulation (rTMS) of the dorsolateral prefrontal cortex (DLPFC) with olfactory stimulation designed to be pleasurable. The present study adhered to rigorous standards for participant inclusion and grouping, encompassing a comprehensive evaluation of clinical and outcome measures. Standardization of rTMS and olfactory stimulation protocols was implemented, ensuring the rigor of the experimental design. Ethical safety guidelines were scrupulously followed to ensure the well-being of the subjects. This approach provides a foundation for the non-invasive treatment of MDD and holds promise for enhancing dopamine release, facilitating a more thorough exploration of the pathophysiological mechanisms underlying MDD.

The small-world network model is a significant indicator of the brain’s topological structure. Zhou and Long conducted a systematic review and found that, in healthy women, the small-world network model of brain functional networks tends to show regularization, while in men, it tends toward randomization. However, the findings do not provide a definitive conclusion regarding structural networks, thereby suggesting the potential importance of gender differences in brain development. However, the paucity of research on mental illness populations has precluded the confirmation of whether gender differences exist in the brain topology of psychiatric patients, which may hinder the development of personalized treatment. Consequently, future research should prioritize the study of psychiatric patients to elucidate gender disparities in brain networks and furnish clinical evidence for personalized, precise treatments.
